# RESIDUAL GASTRIC VOLUME IN MORBIDLY OBESE DIABETICS AFTER AN OVERNIGHT FASTING OR 3 HOURS OF A CARBOHYDRATE-ENRICHED SUPPLEMENT: A RANDOMIZED CROSSOVER PILOT STUDY

**DOI:** 10.1590/0102-672020230073e1791

**Published:** 2024-02-05

**Authors:** Gunther Peres PIMENTA, Ozgur DANDIN, Cervantes CAPOROSSI, José Eduardo AGUILAR NASCIMENTO

**Affiliations:** 1 Universidade de Varzea Grande, Department of Surgery – Varzea Grande (MT), Brazil;; 2 University of Miami, Surgery – Miami (FL), USA.

**Keywords:** Gastric emptying, Carbohydrates, Obesity, Morbid, Diabetes Mellitus Type 2, Esvaziamento Gástrico, Carboidratos, Obesidade Mórbida, Diabetes Mellitus Tipo 2

## Abstract

**BACKGROUND:**

To reduce the risk of regurgitation during anesthesia for elective procedures, residual gastric volumes (RGV) have traditionally been minimized by overnight fasting. Prolonged preoperative fasting presents some adverse consequences and has been abandoned for most surgical procedures, except for obese and/or diabetic patients.

**AIMS:**

The aim of this study was to assess the RGV in morbidly obese diabetic patients after traditional or abbreviated fasting.

**METHODS:**

This study was approved by the Ethics Committee for Research with Human Beings from the Federal University of Mato Grosso, under number 179.017/2012. This is a prospective, randomized, and crossover design study in eight morbidly obese type II diabetic patients. RGV was measured endoscopically after either traditional overnight fasting of at least 8 hours, or after abbreviated fasting of 6 hours for solids and 3 hours for a drink containing water plus 25 g (12.5%) of maltodextrin. Data were expressed as mean and range and differences were compared with paired *t*-tests at p<0.05.

**RESULTS:**

The study population had a mean age of 41.5 years (28–53), weight of 135 kg (113–196), body mass index of 48.2 kg/m^2^ (40–62.4), and type II diabetes for 4.5 years (1–10). The RGV after abbreviated fasting was 21.5 ml (5–40) vs 26.3 ml (7–65) after traditional fasting. This difference was not significant (p=0.82).

**CONCLUSIONS:**

Gastric emptying in morbidly obese diabetic patients is similar after either traditional or abbreviated fasting with a carbohydrate drink.

## RESUMO

**RACIONAL:**

Para reduzir o risco de regurgitação durante a anestesia para procedimentos eletivos, os volumes gástricos residuais (VRG) têm sido tradicionalmente minimizados com jejum noturno. O jejum pré-operatório prolongado tem algumas consequências adversas e tem sido abandonado para a maioria dos procedimentos cirúrgicos, com exceção de pacientes obesos e/ou diabéticos.

**OBJETIVOS:**

O objetivo deste estudo foi avaliar o VRG em pacientes diabéticos obesos mórbidos após jejum tradicional ou abreviado.

**MÉTODOS:**

Este estudo foi aprovado pelo Comitê de Ética em Pesquisa com Seres Humanos da Universidade Federal de Mato Grosso, sob o número 179.017/2012. Este é um projeto prospectivo, randomizado e cruzado em 8 pacientes diabéticos tipo II com obesidade mórbida. O VRG foi medido endoscopicamente após jejum noturno tradicional (pelo menos 8 horas) ou após jejum abreviado (6 horas para sólidos e 3 horas para uma bebida contendo água mais 25 g (12,5%) de maltodextrina). Os dados são expressos como média e variação e as diferenças foram comparadas com testes t pareados em p<0,05.

**RESULTADOS:**

A população estudada tinha 41,5 (28–53) anos de idade, peso médio de 135 (113–196) kg, índice de massa corporal (IMC) de 48,2 (40–62,4) kg/m^2^ e diabetes tipo II de 4,5 (1–10) anos. O VRG após o jejum abreviado foi de 21,5 (5–40) ml versus 26,3 (7–65) ml após o jejum tradicional. Essa diferença não foi significativa (p=0,82).

**CONCLUSÕES:**

O esvaziamento gástrico em pacientes diabéticos obesos mórbidos é semelhante após jejum tradicional ou abreviado com uma bebida com carboidrato.

## INTRODUCTION

Preoperative overnight fasting for elective surgical patients ensures safe gastric emptying and is prescribed to avoid complications, such as vomiting and aspiration of gastric contents, during anesthesia induction or light sedation^26,29,42^. However, this protocol was instituted in the middle of the last century when anesthetic techniques were rudimentary^42^.

It is now recognized that prolonged fasting can amplify the organic response to the trauma of surgery by increasing insulin resistance, and acute-phase response, and also it may lead to an increased loss of lean body mass^14,31^. By the 1980s, it was already known that gastric emptying of water and other non-caloric fluids followed an extremely fast exponential curve^12,15^. A large body of evidence has consistently shown that oral intake of clear fluids up to 2 hours before the induction of anesthesia does not increase gastric volume or acidity^4,27,30,35^. Furthermore, carbohydrate (CHO)-rich drinks given 2–3 hours prior to the anesthesia induction may add beneficial effects for patients. The intake of CHO-rich drinks not only is safe^11,32^ but also may decrease postoperative insulin resistance^15^ and reduce nausea and vomiting^13,20^. Thus, it may enhance postoperative recovery. For these reasons, many services have abandoned antiquated policies of prolonged fasting in most conditions^23,25,28,31^, except for intestinal obstruction, obesity, and presumed slow gastric emptying conditions such as pregnancy, diabetes, and gastroparesis^2,4,33,37^. Various international societies of anesthesia recommend 6–8 hours fasting for solids but allow clear or CHO-drinks 2–3 hours before surgery^21,23,26,43^.

Morbidly obese patients have increased intra-abdominal pressure, and consequently, higher rates of gastroesophageal reflux^24,41^. However, some studies have shown that there is no difference in the time required for gastric emptying in obese individuals, compared with non-obese ones^28,36,44^. Nonetheless, there are few studies that investigated the RGV 2–3 hous after the intake of CHO-drink in obese diabetic patients. New data on this topic would be important to increase the debate on whether it is safe or not to abbreviate preoperative fasting in this subset of patients. Therefore, the aim of this study was to assess the RGV in morbidly obese type II diabetic patients by examining gastroscopy after either traditional fasting or abbreviated fasting with CHO-rich drinks.

## METHODS

This is a prospective, randomized, and crossover trial design with a group of morbidly obese and type II diabetic patients. This study was approved by the Ethics Committee for Research with Human Beings from the Federal University of Mato Grosso, under 179.017/2012; it is in accordance with the ethics principles set out in the Helsinki Declaration (2000) and meets the Brazilian national legal specifications. All participants signed an informed consent form. The study was registered on clinicaltrails.gov under the number NCT02114008.

The population of this study was composed of eight type II diabetic patients with morbid obesity. These obese patients were all on the waiting list as candidates for bariatric procedures. We included those who were between 18 and 55 years of age, of both sexes, with body mass index equal to or greater than 40 kg/m^2^. They were all using either oral hypoglycemics or insulin to control the diabetes. The diabetes in all participants was under control (fasting glycemia less than 140 mg/dL) and an endocrinologist provided information that the amount of maltodextrin intake would not induce any harm to them. No subjects received additional antidiabetic drugs before or after the intake of the CHO-rich drink. We excluded patients with gastroparesis, with prior abdominal operations, those who did not follow the fasting protocol, and who were using medications such as prokinetics and H^+^ pump inhibitors for gastrointestinal symptoms.

The randomization was done at admission through numbers generated by a computer software available online at www.graphpad.com.

Patients underwent two endoscopic examinations at a two-week interval. The main endpoint was the RGV measured by a certified endoscopist after aspiration of gastric contents into a graduated cup.

We compared the RGV after either traditional fasting of at least 8 hours before the test or abbreviated fasting of 6 hours fasting for solids followed by the intake of 200 ml of a beverage containing water plus 25 g (12.5%) maltodextrin 3 hours before the test.

All endoscopic procedures were scheduled to begin at 9 AM and were conducted by the same board-certified endoscopist, who performed blind to the study design. The endoscopies were conducted at Gastroclinica, Cuiaba, Brazil, between April and June 2018. Sedation was performed by a certified anesthesiologist who was also blind to this study. An intravenous bolus injection of 2 ml of lidocaine hydrochloride (Astra Zeneca, São Paulo, Brazil) followed by 100 to 150 mg of propofol before endoscopy were done in all cases. Digital oximetry was carried out throughout the procedure. Patients were positioned in left lateral recumbent throughout the endoscopy. A flexible electronic video endoscope (EG2770K; Pentax Corporation, Tokyo, Japan) of 9 mm in outer diameter was used for conventional upper endoscopy, according to a standard protocol recommended by the fabricator. All gastric fluid was thoroughly suctioned through an endoscope side port. The RGVs were measured and recorded by the endoscopist after aspiration of the gastric contents.

A comparison of RGV between the two groups was done by paired *t*-tests. All statistical analyses were conducted using the software Statistical Package for Social Sciences (SPSS for Windows 11.0). The minimum accepted significance level was 5% (p<0.05).

## RESULTS

All participants followed the fasting protocol in the two periods of the study. There were no complications during the endoscopic procedures. The gastroscopist reported that in all cases, the aspiration of gastric contents was very easy and did not extend the duration of the procedure. All patients were found to have mild gastritis.

All patients were receiving oral hypoglycemic drugs, and two of them were under different doses of subcutaneous insulin. All had fasting hyperglycemia less than or equal to 140 mg/dL at the moment of the test. The clinical and demographic characteristics of the participants can be seen in Table 1.

**Table 1:** Demographic characteristics of the patients.

Variable	Number or median	Range or %
Age (y/o)	41	28–53
Male (n, %)	4	50
Weight (kg)	136	113–196
BMI (kg/m^2^)	48.2	40.1–62.1
Time having diabetes (y)	4.5	3–10

BMI: body mass index.

**Figure 1:**
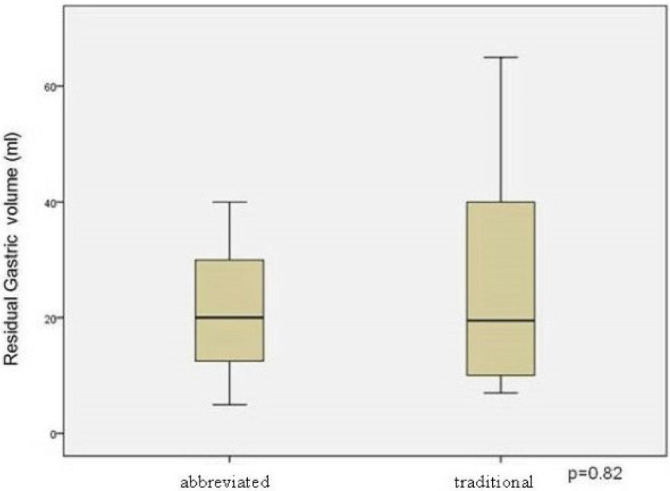
Residual gastric volume after abbreviated and traditional fasting.

Gastric contents were found in all patients. The mean RGV in the abbreviated fasting group was 21.5 ml (range 5–40 ml) while in the traditional fasting group, it was 26.3 ml (range 7–65 ml). These findings are illustrated in Figure 1.

The difference between the groups was not significant (p=0.82). The RGVs of each one of the patients are shown in Table 2.

**Table 2:** Residual gastric volume (mL) found in each individual after traditional or abbreviated fasting.

Patient number	Abbreviated Fasting (3h CHO-drink)	Traditional Fasting (8h)
1	5	65
2	10	14
3	20	10
4	20	7
5	40	40
6	20	25
7	40	40
8	15	10

CHO: carbohydrate. p=0.82 (paired *t*-test)

## DISCUSSION

The main finding of the study was that CHO-rich drink given 3 hours before sedation for endoscopic procedure did not increase the RGV in this group of morbidly obese diabetic patients. Another important finding was that all exams were easily performed without anesthetic complication such as gastroesophageal reflux or broncho aspiration. These results suggest that the abbreviation of fasting for upper endoscopy in morbidly obese patients is safe. Furthermore, these findings not only reinforce the prescription of CHO-rich drinks before procedures to abbreviate fasting, but they are also in accordance with previous data from the literature^2,5,33^.

Many traditional peri-procedural prescriptions, including pre-procedural overnight fasting, are based on dogma rather than scientific evidence^19,29^. Supported by the dogma of “nil per os” before surgery^29^, obese patients have been considered a high-risk population for bronchial aspiration during the induction of anesthesia due to a combination of increased gastric acid secretion and low gastric pH^40^. However, it was shown that the intake of clear liquid or CHO-rich drinks 2–3 hours before gastroscopy did not elevate the gastric pH nor increase the RGV^3,38^. Another study showed that gastric emptying in patients with type II diabetes is not delayed, suggesting that a CHO-rich beverage may be safely administered before anesthesia^18^. Finally, studies have shown that fasting abbreviation in morbidly obese patients undergoing sleeve gastrectomy is safe^22,34^.

Modern procedures recommended by multimodal protocols^1,17,37,43^ in elective surgical patients include the reduction of preoperative fasting times, which may result in reduced morbidity and shorter postoperative hospital stays^9,34,39^. Prolonged preoperative fasting induces insulin resistance, stimulates glycogenolysis, proteolysis, and lipolysis, and may increase glycemia^7,10^. Insulin resistance induced by fasting is similar to the mechanism occurring in patients with type II diabetes^16^. This is followed by a decrease in oxidative glucose disposal, particularly in muscle cells^6^. Prolonged preoperative fasting may also lead to an increase in the inflammatory markers of the acute-phase response after surgery. Conversely, CHO-rich oral supplements taken 2–3 hours before surgery may attenuate the metabolic response^37,41,45^. The meta-analyses conducted by Awad et al., including 21 controlled and randomized studies, concluded that the abbreviation of preoperative fasting in major elective abdominal surgeries may reduce the length of hospital stay and postoperative insulin resistance^8^.

One of the greatest current challenges is the implementation of a protocol with 2–3 hour fasting period. This is because, in addition to the problem of dogma, it is easier to simply prescribe no oral intake after midnight. Therefore, it is difficult to educate patients and hospital staff to adhere to the new guidelines^26^ and implement fasting abbreviation.

Despite the advantages and safety demonstrated by new studies^2,5,33,37,43,44^, as well as in this study, there is still a need for more scientific evidence. The findings in our pilot study warrant some criticism, as the sample size was very small, limited to only 8 individuals. Thus, extrapolation of these findings for clinical application should be done with caution. However, crossover works are considered adequate and valid in the current literature to address this type of research question. Controlled randomized crossover works hold significant importance in medical research. This design enables the subjects to be compared with themselves, guaranteeing optimal control groups. As a result, this significantly reduces biases and confounding variables compared to other randomized studies. Furthermore, we can conclude that crossover works are statistically efficient, cost-effective, and require only a small number of individuals in their design^25^.

## CONCLUSIONS

Gastric emptying in morbidly obese diabetic patients is similar after either traditional or abbreviated fasting with a carbohydrate-rich drink. These findings support the idea of shortening the preoperative fasting period for sedation or general anesthesia in obese patients with type II diabetes.

## References

[1] Aguilar-Nascimento JE, Bicudo-Salomao A, Caporossi C, Silva RM, Cardoso EA, Santos TP (2008). Enhancing surgical recovery in Central-West Brazil: the ACERTO protocol results.. E Spen Eur E J Clin Nutr Metab..

[2] Nascimento JEA, Campos AC, Borges A, Correia MITD (2011). Tavares GMProjeto Diretrizes;. Terapia nutricional no perioperatório. São Paulo: Sociedade Brasileira de Nutrição Parenteral e Enteral..

[3] Aguilar-Nascimento JE, Caporossi C, Metelo JS, Tanajura GH, Canevari-de-Oliveira M, Costa RC (2014). Safe intake of na oral supplement containing carbohydrates and whey protein shortly before sedation to gastroscopy; a double blind, randomized trail.. Nutr Hosp..

[4] Aguilar-Nascimento JE, Dock-Nascimento DB (2010). Reducing preoperative fasting time: a trend based on evidence.. World J Gastrointest Surg..

[5] American Society of Anesthesiologists Committee (2011). Practice guidelines for preoperative fasting and the use of pharmacologic agents to reduce the risk of pulmonary aspiration: application to healthy patients undergoing elective procedures: an updated report by the American Society of Anesthesiologists Committee on Standards and Practice Parameters.. Anesthesiology..

[6] Awad S, Constantin-Teodosiu D, Macdonald IA, Lobo DN (2009). Short-term starvation and mitochondrial dysfunction – a possible mechanism leading to postoperative insulin resistance.. Clin Nutr..

[7] Awad S, Stephenson MC, Placidi E, Marciani L, Constantin-Teodosiu D, Gowland PA (2010). The effects of fasting and refeedindg with a ‘metabolic preconditioning’ drink on substrate reserves and mononuclear cell mitochondrial function.. Clin Nutr..

[8] Awad S, Varadhan KK, Ljungqvist O, Lobo DN (2013). A meta-analysis of randomised controlled trials on preoperative oral carbohydrate treatment in elective surgery.. Clin Nutr..

[9] Bicudo-Salomão A, Meireles MB, Caporossi C, Crotti PL, Aguilar-Nascimento JE (2011). Impact of the ACERTO project in the postoperative morbi-mortality in a university hospital.. Rev Col Bras Cir..

[10] Blixt C, Ahlstedt C, Ljungqvist O, Isaksson B, Kalman S, Rooyackers O (2012). The effect of perioperative glucose control on postoperative insulin resistance.. Clin Nutr..

[11] Brady M, Kinn S, Stuart P (2003). Preoperative fasting for adults to prevent perioperative complications.. Cochrane Database Syst Rev..

[12] Brener W, Hendrix TR, McHugh PR (1983). Regulation of the gastric emptying of glucose.. Gastroenterology..

[13] Carlos LO, Ramos MRZ, Wagner NRF, Freitas LAC, Felicidade I, Campos ACL (2022). Probiotic supplementation attenuates binge eating and food addiction 1 year after roux-en-y gastric bypass: a randomized, double-blind, placebo-controlled trial.. Arq Bras Cir Dig..

[14] De-Aguilar-Nascimento JE, Salomão AB, Waitzberg DL, Dock-Nascimento DB, Correa MITD, Campos ACL (2017). ACERTO guidelines of perioperative nutritional interventions in elective general surgery.. Rev Col Bras Cir..

[15] Erskine L, Hunt JN (1981). The gastric emptying of small volumes given in quick succession.. J Physiol.

[16] Faria MS, Aguilar-Nascimento JE, Pimenta OS, Alvarenga LC, Dock-Nascimento DB, Slhessarenko N (2009). Preoperative fasting of 2 hours minimizes insulin resistance and organic response to trauma after video-cholecystectomy: a randomized, controlled, clinical trial.. World J Surg.

[17] Fearon KC, Ljungqvist O, Von Meyenfeldt M, Revhaug A, Dejong CH, Lassen K (2005). Enhanced recovery after surgery: a consensus review of clinical care for patients undergoing colonic resection.. Clin Nutr..

[18] Greenfield SM, Webster GJ, Brar AS, Ah Mun K, Beck ER, Vicary FR (1996). Assessment of residual gastric volume and thirst in patients who drink before gastroscopy.. Gut..

[19] Gustafsson UO, Nygren J, Thorell A, Soop M, Hellström PM, Ljungqvist O (2008). Pre-operative carbohydrate loading may be used in type 2 diabetes patients.. Acta Anaesthesiol Scand..

[20] Hausel J, Nygren J, Thorell A, Lagerkranser M, Ljungqvist O (2005). Randomized clinical trial of the effects of oral preoperative carbohydrates on postoperative nausea and vomiting after laparoscopic cholecystectomy.. Br J Surg.

[21] Kuemmerli C, Tschuor C, Kasai M, Alseidi AA, Balzano G, Bouwense S (2022). Impact of enhanced recovery protocols after pancreatoduodenectomy: meta-analysis.. Br J Surg..

[22] Lemanu DP, Singh PP, Berridge K, Burr C, Barbor R, MacCormick AD (2013). Randomizeed clinical trial of enhanced recovery versus standard care after laparoscopic sleeve gastrectomy.. Br J Surg..

[23] Ludwig RB, Paludo J, Fernandes D, Scherer F (2013). Lesser time of preoperative fasting and early postoperative feeding are safe?. Arq Bras Cir Dig..

[24] Luna RA, Peixoto EM, Carvalho CFA, Velasque LS (2022). Impact of body mass index on perioperative outcomes for complex hiatus hernia by videolaparoscopy.. Arq Bras Cir Dig..

[25] Maclure M, Mittleman MA (2000). Should we use a case-crossover design?. Annu Rev Public Health..

[26] Maltby JR (2006). Fasting from midnight--the history behind the dogma.. Best Pract Res Clin Anaesthesiol..

[27] Maltby JR, Lewis P, Martin A, Sutherland LR (1991). Gastric fluid volume and pH in elective patients following unrestricted oral fluid until three hours before surgery.. Can J Anaesth..

[28] Maltby JR, Pytka S, Watson NC, Cowan RA, Fick GH (2004). Drinking 300ml of clear fluid two hours before surgery has no effect on gastric fluid volume and ph is fasting and non-fasting obese patients.. Can J Anaesth.

[29] Mendelson CL (1946). The aspiration of stomach contents into the lungs during obstetric anesthesia.. Am J Obst Gynecol..

[30] Nogueira PLB, Silva MR, Dock-Nascimento DB, Aguilar-Nascimento JE (2022). Residual gastric volume after 3 h of the ingestion of an oral supplement containing carbohydrates alone or associated with whey protein: a randomized crossover pilot study.. Perioper Med (Lond)..

[31] Oliveira KG, Balsan M, Oliveira SS, Aguilar-Nascimento JE (2009). Does abbreviation of preoperative fasting to two hours with carbohydrates increase the anesthetic risk?. Rev Bras Anestesiol..

[32] Nygren J (2006). The metabolic effects of fasting and surgery.. Best Pract Res Clin Anaesthesiol..

[33] Pimenta GP, Capellan DA, Aguilar-Nascimento JE (2015). Sleeve gastrectomy with or without a multimodal perioperative care.. A randomized pilot study. Obes Surg..

[34] Pimenta GP, Aguilar-Nascimento JE (2014). Prolonged preoperative fasting in elective surgical patients: why should we reduce it?. Nutr Clin Pract..

[35] Phillips S, Hutchinson S, Davidson T (1993). Preoperative drinking does not affect gastric contents.. Br J Anaesth..

[36] Reis LA, Reis GFF, Oliveira MRM (2010). The airway and gastric contents in obese patients.. Rev Bras Anestesiol..

[37] Singh SM, Liverpool A, Romeiser JL, Miller JD, Thacker J, Gan TJ (2021). A U.S. survey of pre-operative carbohydrate-containing beverage use in colorectal enhanced recovery after surgery (ERAS) programs.. Perioper Med (Lond)..

[38] Unal Y, Sarioglu M, Korkmaz T (2009). Effects on gastric residue and ph of fasting period before gastroscopy.. Gazi Med J..

[39] Varadhan KK, Neal KR, Dejong CHC, Fearon KCH, Ljungqvist O, Lobo DN (2010). The enhanced recovery after surgery (ERAS) pathway for patients undergoing major elective open colorectal surgery: a meta-analysis of randomized controlled trials.. Clin Nutr..

[40] Vaughan RW, Bauer S, Wise L (1975). Volume and ph of gastric juice in obese patients.. Anesthesiology..

[41] Viganò J, Cereda E, Caccialanza R, Carini R, Cameletti B, Spampinato M (2012). Effects of preoperative oral carbohydrate supplementation on postoperative metabolic stress response of patients undergoing elective abdominal surgery.. World J Surg..

[42] Warner MA (2000). Is pulmonary aspiration still an import problem in anesthesia?. Curr Opin Anesthesiol..

[43] Weimann A, Braga M, Carli F, Higashiguchi T, Hübner M, Klek S (2021). ESPEN practical guideline: clinical nutrition in surgery.. Clin Nutr..

[44] Wong CA, McCarthy RJ, Fitzgerald PC, Raikoff K, Avram MJ (2007). Gastric emptying of water in obese pregmant women at term.. Anesth Analg..

[45] Zelić M, Stimac D, Mendrila D, Tokmadžić VS, Fišić E, Uravić M (2012). Influence of preoperative oral feeding on stress response after resection for colon cancer.. Hepatogastroenterology..

